# Revealing the Disturbed Vaginal Micobiota Caused by Cervical Cancer Using High-Throughput Sequencing Technology

**DOI:** 10.3389/fcimb.2020.538336

**Published:** 2020-12-07

**Authors:** Yupei Xie, Ying Feng, Wenyu Li, Fuliang Zhan, Genhua Huang, Hui Hu, Yifei Xiong, Buzhen Tan, Tingtao Chen

**Affiliations:** ^1^ Department of Obstetrics and Gynecology, The Second Affiliated Hospital of Nanchang University, Nanchang, China; ^2^ National Engineering Research Center for Bioengineering Drugs and the Technologies, Institute of Translational Medicine, Nanchang University, Nanchang, China

**Keywords:** vaginal microbiota, cervical cancer, cervical squamous intraepithelial neoplasia, *Lactobacillus*, vaginal microbial disorders

## Abstract

Cervical cancer is the fourth most prevalent cancer type among all malignancies, so it is of great significance to find its actual pathogenesis mechanisms. In the present study, 90 women were enrolled, and high-throughput sequencing technology was firstly used to analyze the vaginal microbiota of healthy women (C group), cervical intraepithelial neoplasia patients (CIN group) and cervical cancer patients (CER group). Our results indicates that compared with C group, a higher HPV infection rate as well as increased Neutrophil ratio and tumor marker squamous cell carcinoma antigen (SCCA) were obtained, and a decrease in Lymphocyte ratio and Hemoglobin were also present. In addition, the cervical cancer showed a strong association with reduced probiotics *Lactobacillus*, increased pathogens *Prevotella* spp., *Sneathia* spp. and *Pseudomonas* spp. These results prove that the immunological changes generated by the cervical cancer and the vaginal microbiota can interact with each other. However, further study investigating the key bacteria for cervical cancer is still needed, which can be a clue for the diagnosis or treatment of cervical cancer.

## Introduction

Cervical cancer belongs to the most common gynecological malignancies, causing 570,000 new cases and 311,000 deaths worldwide in 2018 (Bray et al., 2018). According to international federation of gynecology and obstetrics (FIGO) 2018 guidelines, surgery and radiation therapy are the main treatments, and chemotherapy is the main adjuvant treatment at present (Bhatla et al., 2018). However, complications such as the nerve damage or bladder function damage caused by surgery (Koh et al., 2019), and the destroyed immune system, enteritis and vaginitis caused by radiotherapy (Hosaka and Watari, 2012) are also present, which greatly hinder the patients’ compliance during the period of treatment. Therefore, figuring out an adjuvant therapy to enhance the efficacy or reduce the side effect of the current treatments for cervical cancer is urgently needed.

In previous studies, a strong connection between cervical human papillomavirus (HPV) infection and cervical cancer has been identified, and HPV infected cell could express tumorigenic protein E6 and E7, which might further suppress the expression of tumor suppressor p53 and pRb (Oyervides-Munoz et al., 2018). However, most HPV infections could be eliminated by the immune system in a short time, and only a small percentage of women’s immune systems failed to clear the infected HPV, which lead to its integration into the host genome, causing malignancy of the uterus cervix (Munger et al., 2004; Senapati et al., 2016). Moreover, most patients with low grade cervical intraepithelial lesion might not eventually develop into invasive cervical cancer (Nam et al., 2009; Senapati et al., 2016). As a result, future studies are necessary to validate the contributing factors for cervical cancer.

As one of the four major bacteria stores, vaginal microorganism plays an important role in resisting pathogen infection and maintaining the normal environment of the vagina (Romero et al., 2014). Previous research confirmed that *Lactobacillus* was the dominant genus in vaginal tract, with its produced lactic acid, H_2_O_2_ and other acidic items maintaining an acid environment (pH ranged from 3.5–4.5) in vaginal tract to defend pathogens, and to stimulate the host *via* producing the cytokines that inhibit inflammation (Hearps et al., 2017). Studies indicated that vaginal microbiota might be strongly affected by the physiological anatomy of the genitals, the endocrine function and immune system of human body (Romero et al., 2014). The reduction of probiotics *Lactobacillus* and the overgrowth of anaerobic bacteria (e.g. *Gardnerella vaginalis*, *Morbiluncus* spp., *Prevostella* spp., *Mycoplasma hominis* and *Atopobium vaginae*) could significantly increase the risk of sexually transmitted infections (Monin et al., 2019). Moreover, the imbalance of vaginal microbiota could also cause female reproductive tract inflammation (Martin, 2012) and adverse pregnancy outcomes (Seale et al., 2016), which might seriously affect the occurrence and development of cervical cancer (Kwasniewski et al., 2018).

Although prior researches have indicated that vaginal microbes play a critical role in gynecological diseases (e.g. cervical lesions and HPV infections), little work has been done to reveal the potential relationship between vaginal microbiota and cervical cancer. Therefore, 90 volunteers were enrolled in the present study to explore the differences of vaginal microbiota among healthy women, patients with cervical intraepithelial neoplasia and patients with cervical cancer based on high through-put sequencing method.

## Materials and Methods

### Study Groups and Sampling

All patients included in this study were diagnosed for the first time in the Second Affiliated Hospital of Nanchang University, aged 25–39 years (mean age 34.57 ± 2.665). All of them did not use antibiotics within one month, denied smoking or drinking habits, denied sex and vaginal irrigation within 1 week. All patients accepted general tests including blood routine tests, leucorrhea routine tests (shown no fungi infection), liver function tests, renal function tests, treponema pallidum, human immunodeficiency virus (HIV) screening, herpes simplex virus (HSV) screening, HPV typing test, liquid-based pathological examination of cervical exfoliated cells and pelvic examination (gynecological examination) in the Second Affiliated Hospital of Nanchang University and then an inspection report was finally issued.

Volunteers were divided into C group (negative for combined test of HPV screening and TCT screening, N = 30), CIN group (abnormalities for HPV screening or TCT screening, and cervical pathological biopsy suggests cervical intraepithelial neoplasia, N = 30) and CER group (cervical cancer patients diagnosed by TCT screening, HPV screening and cervical biopsy, N = 30). Before the pelvic examination, the sterile disposable cotton swab was used to collect vaginal secretions from the posterior vaginal fornix, which were then stored in a −80°C refrigerator for further use (Virtanen et al., 2019).

This study has been reviewed and approved by Ethics committee of the Second Affiliated Hospital of Nanchang University. All patients participating in this clinical study have been informed in advance, and consent was signed before the collection of vaginal secretions. This study has been registered and approved by the China Clinical Trial Registry (No. ChiCTR1900018419).

### HPV Subtypes Detection and Cervical Cytology Test

HPV samples were obtained by brushing the cervical canal during the pelvic examination, and polymerase chain reaction reverse dot blot hybridization (PCR-RBD) was used to detect HPV virus in cervical samples (Sun et al., 2017; Zhang Z. et al., 2018). The PCR method is used to amplify the DNA fragments extracted from the samples, and the PCR product of about 200 bp was further mixed and reacted with the HPV DNA chip and its related reaction solution, and the result was interpreted after color development, so as to realize the detection of HPV type. HPV detection kit used in this experiment was purchased from Guangzhou LBP Medical Technology Co., Ltd. A total of 28 HPV subtypes were tested in this experiment, including high-risk types 16, 18, 26, 31, 33, 35, 39, 45, 51, 52, 53, 56, 58, 59, 66, 68, 73, 82; and low-risk type 6, 11, 40, 42, 43, 44, 54, 61, 81, 83.

Cytopathological examination of cervical exfoliated cells were carried out using the Thin PrepPap test method which is approved by the US Food and Drug Administration (FDA) in 1996 (Wilbur, 2003). Liquid-based cell treatment reagents was added to the obtained cervical exfoliated cells to lyse the red blood cells in the sample to avoid interference with the experimental results. At the same time, the fixed components in the reagent could preserve valuable cells such as fixed white blood cells and exfoliated epithelial cells; and fully separate the effective cells wrapped in mucus to prevent the loss of valuable cells. After the smearing, the sample was stained with Pap staining. After that, two cytopathologists observed under an electron microscope to make a diagnosis. Cytopathology reports were divided into the following five categories: 1, No intraepithelial lesions and malignant lesions (NILM); 2, Low-grade squamous intraepithelial lesion (LSIL); 3, Atypical squamous cell (ASC); 4, High-grade squamous intraepithelial lesion (HSIL); 5, Cervical cancer.

### Bacterial Genomic DNA Extraction and High-Throughput Sequencing

The bacterial genomic DNA extraction kit (Tiangen Biotech Co., Ltd., Beijing, China) and bead beating method were used in combination to extract bacterial genomic DNA, and then the concentration and purity of the extracted DNA were determined using a spectrophotometer at 230 nm (A 230) and 260 nm (A 260) (NanoDrop; Thermo Fisher Scientific, Inc., Waltham, MA, USA). The V4 region of 16 srDNA in each sample was amplified using primers 520F/802R (520F, 5′-AYTGGGYDTAAAGNG-3′; 802R, 5′-AYTGGGYDTAAAGNG-3′), (GenBank accession number PRJNA595048). The PCR products were sequenced by using the Illumina Novaseq platform sequencer in Personal Biotechnology Co., Ltd., Shanghai, China.

**Figure 1:**
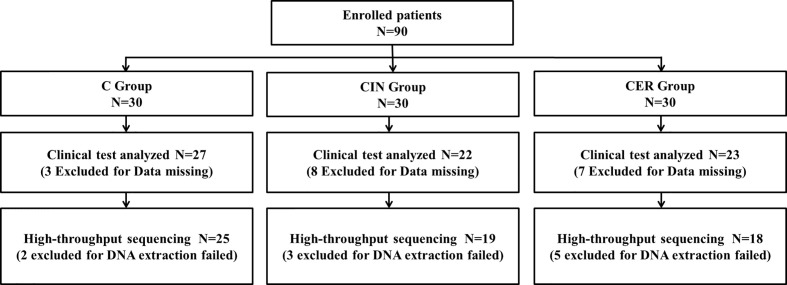
This study initially included 90 volunteers, of which 18 were excluded due to lack of clinical test data (3 in group C, 8 in CIN group, and 7 in CER group). Thereafter, the vaginal posterior fornix secretions of these 72 volunteers were collected for bacterial and microbial DNA extraction. During this process, 10 volunteers failed to extract DNA and were excluded (2 in group C, 3 in CIN group and 5 in the CER group).

As shown in **Figure 1**, the total of 90 women were initially included in the study during July 2018 to August 2019, of which 30 were normal women (C group), 30 were patients with cervical intraepithelial lesions (CIN group) and 30 were cervical cancer patients (CER group). Then, 18 women were excluded due to incomplete clinical test data (3 in C group; 8 in CIN group and 7 in CER group), and 10 women were further excluded before high-throughput sequencing due to microbiota genomic DNA extraction failure (2 in C group; 3 in CIN group and 5 in CER group). The rest 72 volunteers’ vaginal microbiome was finally analyzed. Using the degree of cervical lesions as a confounding factor, a stratified analysis on the correlation between vaginal microbes and blood biochemical indicators was performed. The cervical cancer patients were divided into two groups according to the FIGO2018 staging standard (Koh et al., 2019): stage earlier than Ib1 (n = 6) and stage later than or equal to Ib1 (n = 12). Patients with cervical precancerous lesions were divided into three degrees: lesions infiltration less than 1/3, CINI (n = 1); lesion infiltration is greater than 1/3 and less than 2/3, CINII (n = 7); Lesions infiltrate more than 2/3 but do not penetrate the substrate, CINIII (n = 11).

### Vaginal Bacterial Diversity Analyzing

This project uses the Illumina MiSeq platform to perform paired-end sequencing of community DNA fragments and used sliding window method to screen the quality of the two-end sequences one by one. Subsequently, using the FLASH software (v1.2.7 http://ccb.jhu.edu/software/FLASH/) (Magoč and Salzberg, 2011), the paired-end sequences that passed the preliminary quality screening were paired and ligated according to overlapping bases, and the connected sequence identification was assigned to the corresponding sample, thereby obtaining a valid sequence for each sample. QIIME software (Quantitative Insights Into Microbial Ecology, v1.8.0, http://qiime.org/) (Caporaso et al., 2010) was used to identify interrogative sequences. VSEARCH (v2.7.1, https://github.com/torognes/vsearch) (Rognes et al., 2016) was then used to check and reject the chimera sequence. Sequences with more than 97% similarity were classified into the same classification operation unit. R software was used to calculate the number of common Operational Taxonomic Units (OTUs) in each sample (group), and the Venn diagram intuitively reflecting the proportion of OTUs common to each sample (group) and the proportion of unique OTUs to each sample (group). The QIIME software (Version 1.8.0) was used to analyze α diversity (include indexes of observed-OTUs, Chao1, Shannon, Simpson, ACE, goods-coverage) and β diversity (include principal component analysis (PCA), principal coordinates analysis (PCoA) and nonmetric Multidimensional Scaling (NMDS)) of the bacteria. The cluster analysis was preceded by weighted UniFrac distance using QIIME software package (version 1.8.0). Theαvalue was set to 0.05 in order to correct multiple comparisons. Finally, we used Phylogenetic Investigation of Communities by Reconstruction of Unobserved States (PICRUST) to analyze the metabolic function and its influence on the host cell of bacteria in Kyoto Encyclopedia of Genes and Genomes (KEGG) functional spectrum database (Liu et al., 2019; Zheng et al., 2019).

### Statistical Analysis

Statistical analyses in this study was performed by using the GraphPad Prism 8 (https://www.graphpad.com) and SPSS 23.0 software (SPSS Inc., Chicago, IL, USA), with data reported as mean ± SD. Statistical significance was determined using a Student’s test, one-way or two-way ANOVA and annotated using the international convention which is related to the statistical representation.

## Result

### Volunteer General Situation Analysis

**Figure 2:**
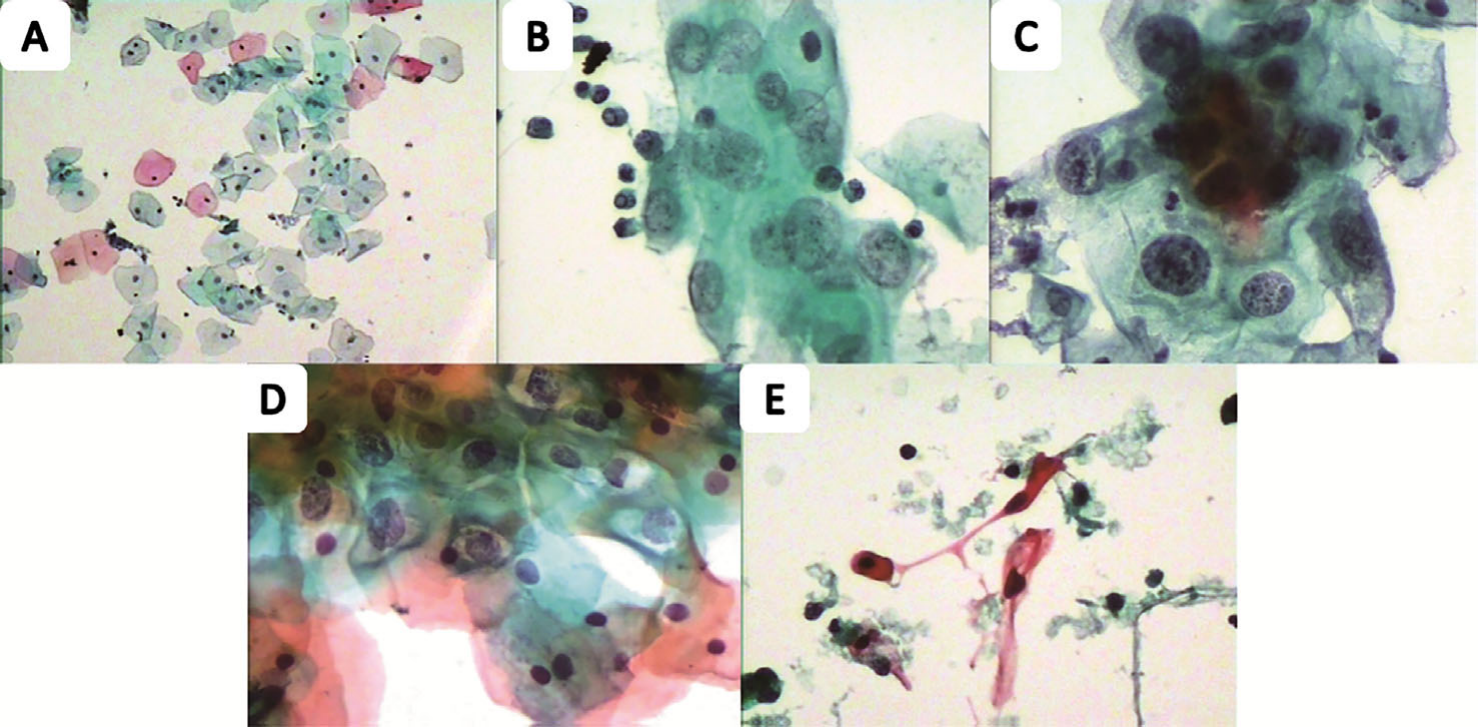
The Pap smear of the cervical cell from five different health status people. The normal one **(A)**, low squamous intraepithelial lesion **(B)**, high squamous intraepithelial lesion **(C)**, atypical squamous cell **(D)** and squamous cell carcinoma **(E)** are from the participants.

We evaluated the age, HPV infection status, and TCT test among C, CIN and CER groups. No significant differences for age were found (p = 0.1374), while in terms of HPV infection status (p<0.001) and TCT test (p<0.001) the differences were statistically significant. In addition, the relationship between HPV infection and TCT test indicated a positive correlation (**Table 1** and **Figure 2**).

**Table 1:** Comparison of patient age, HPV infection status and TCT test results.

Variable	C Group (N = 27)	CIN Group (N = 22)	CER Group (N = 23)	P value
**Percentage of total enrollment,**	27(37.5)	22(30.56)	23(31.94)	/
**No (%).**				
**Age (mean ± std. deviation)**	34.04 ± 3.142	35.53 ± 2.951	34.16 ± 1.914	0.1374
**TCT result category, No (%).**				<0.0001
NILM	27 (100%)	9 (40.91%)	1 (4.34%)	
LSIL	0 (0%)	3 (13.63%)	2 (8.69%)	
HSIL	0 (0%)	5 (22.73%)	9 (39.14%)	
ASC	0 (0%)	5 (22.73%)	7 (30.44%)	
Squamous cell carcinoma	0 (0%)	0 (0%)	4 (17.39%)	
**HPV infection subtype, No (%).**				<0.0001
Negative	27(100%)	2(9.09%)	1(4.35%)	
16 or 18	0(0%)	13(59.09%)	19(82.61%)	
Others	0(0%)	7(31.82%)	3(13.04%)	

Age differences between groups are not statistically significant (P = 0.1347). The rates of high-risk HPV infection and abnormal rates of TCT in the CER and CIN groups were higher than those in the C group (P<0.0001). P value less than 0.05 is statistically significant.

C group, healthy control group; CIN group, Cervical intraepithelial neoplasis group; CER group, Cervical cancer group; NILM, No intraepithelial lesions and malignant lesions; LSIL, low-grade squamous intraepithelial lesion; HSIL, high-grade squamous intraepithelial lesion; ASC, atypical squamous cell.

### Comparison of Immunity and Metabolic Indicators

**Figure 3:**
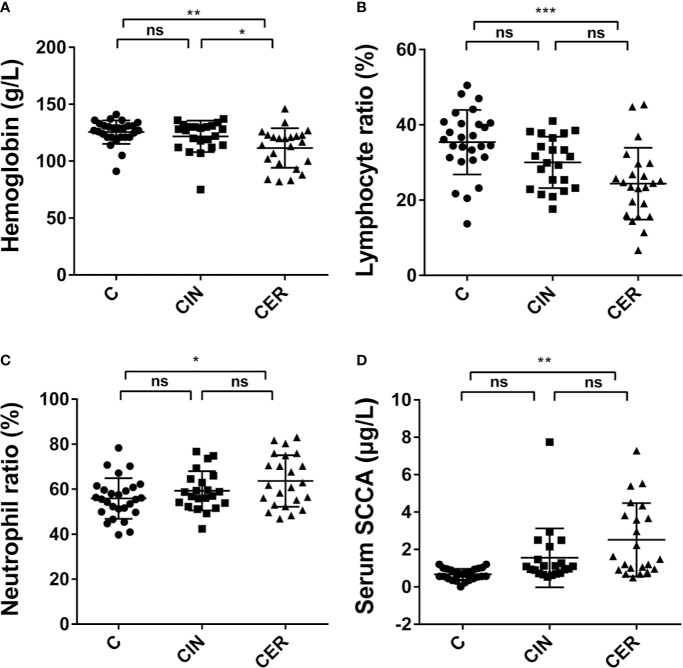
Differences in clinical blood test indicators. In the comparison of neutrophil ratio **(C)** (P = 0.0249) and squamous cell carcinoma antigen (SCCA) **(D)** (P = 0.0001), CER group is higher than CIN group, and CIN group is much higher than C group. To the contrary, the hemoglobin **(A)** (P = 0.0024) and lymphocyte ratio **(B)** (P < 0.0001) in C group is much higher than CIN group and CER group. P value less than 0.05 is statistically significant. * means p < 0.05; * means P < 0.01; ** means P < 0.001; *** means P < 0.001; p < 0.05 indicates significant difference. C, Control group, a group of women who have no problem in gynecology; CER, Cervical cancer group, a group of women who were diagnosed a cervical cancer by pathological biopsy; CIN, Cervical intraepithelial neoplasis group, a group of women who were diagnosed by pathological biopsy or cytological test; ns, not significant.

**Figure 3** shows the results of hematological indicators. Our work indicated that the cervical cancer had significantly changed the Neutrophil ratio (55.84%, 59.26% and 63.60%, respectively. P = 0.0249), Lymphocyte ratio (35.37%, 30.03% and 24.37%, respectively. P<0.0001), Hemoglobin (125.5 g/L, 121.8 g/L, and 111.5 g/L, respectively. P = 0.0024) and the squamous cell carcinoma antigen (SCCA, tumor marker) (0.6674 μg/L, 1.556 μg/L and 2.52 μg/L, respectively. P = 0.0001) in C, CIN and CER groups.

### Vaginal Microbiota Among Groups C, CIN, and CER

**Figure 4:**
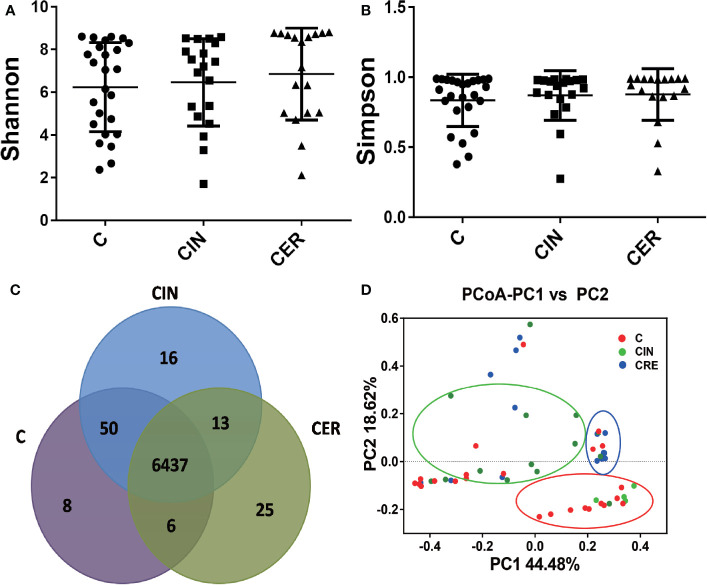
Differences in the alpha diversity and beta diversity of the vaginal flora among these three groups. The Shannon index **(A)** (P = 0.2609), Simpson index **(B)** (P = 0.2245), and Scalar-Venn **(D)** reveal the alpha diversity of the vaginal flora. The difference in alpha diversity has no statistical significance. The Venn result shows that there are 6573 OTUs in the C group, 6516 OUTs in the CIN group and 6517 OUTs in CER group. The PCoA analysis **(C)** reveals the PCoA beta diversity index. C: Control group, a group of women who have no problem in gynecology. CER: Cervical cancer group, a group of women who were diagnosed a cervical cancer by pathological biopsy. CIN: Cervical intraepithelial neoplasis group, a group of women were diagnosed a by pathological biopsy or cytological test.

To explore the microbial differences among groups C, CIN and CER, high-throughput sequencing was used. As shown in **Figure 4**, a higher Shannon index (P = 0.2609) and Simpson index (P = 0.2245) were observed in CIN and CER groups than that in C group. And 6437 common OUTs were obtained, which occupied 99.0%, 98.6% and 99.3% in C, CIN and the CER group, respectively. Moreover, the PCoA analyze indicated that dots in C groups obviously which were deviated from samples in CIN and CER groups.

**Figure 5:**
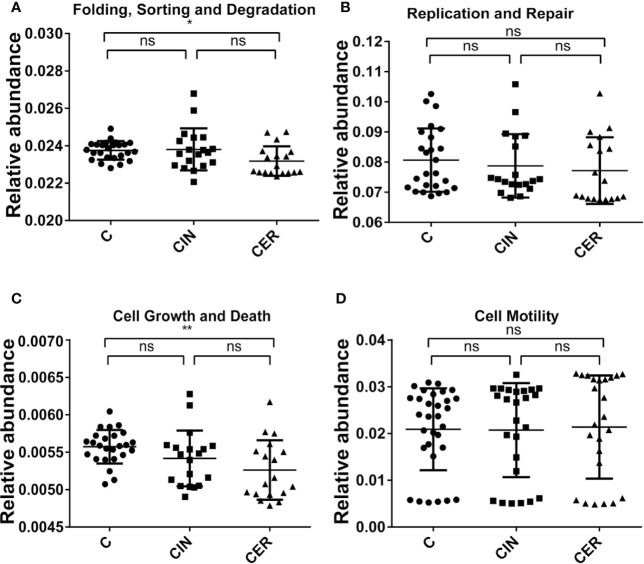
The difference in the construction of the vaginal flora at the level of phylum. The most abundant 20 phyla in each group **(A)**, the differences among the main member **(B)**, and the most common pathogens in the vaginal flora **(C–E)**. C: Control group, a group of women who have no problem in gynecology. CER: Cervical cancer group, a group of women who were diagnosed a cervical cancer by pathological biopsy. CIN: Cervical intraepithelial neoplasis group, a group of women were diagnosed a by pathological biopsy or cytological test. * means p <0.05; ** means P <0.01; *** means P <0.001; p < 0.05 indicates significant difference. ns, no significance.

**Figure 6:**
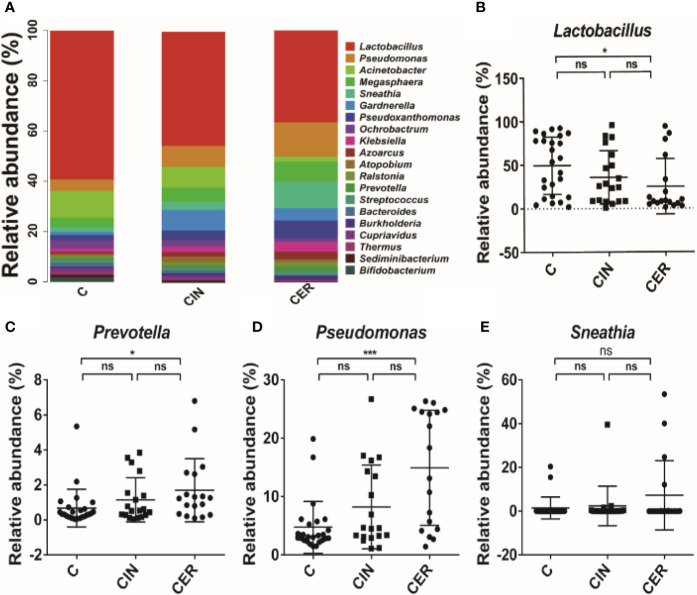
Comparison of the influence on the host cell metabolism and DNA status. Differences in cell growth and death **(A)** (P = 0.0075), and cell motility **(B)** (P = 0.1437), are shown above. As the same, differences in the DNA folding, sorting and degradation **(C)** (P = 0.0184), and DNA replication and repair **(D)** (P = 0.5705). P value less than 0.05 is statistically significant.

Then, we further analyzed the microbial composition at genus level and found that *Lactobacillus* (35.59% in C group, 25.24% in CIN group and 18.42% in CER group, respectively) were the dominant bacteria in C, CIN and CER. As for the probiotics, *Lactobacillus* received a significant reduction in CIN group (25.24%) and CER group (18.42%) than that in C group (35.59%), P = 0.034; while for pathogens, the relative abundance of *Prevotella* spp. (1.15%, 1.70% and 0.68%, respectively, P = 0.029), *Sneathia* spp. (2.37%, 7.27% and 1.45%, respectively, P = 0.176) and *Pseudomonas* spp. (8.22%, 14.92% and 4.75%, respectively, P = 0.0001) were significantly higher in CIN and CER groups than that in C group (**Figure 5**). The present study also indicated that the altered bacterial diversity had significantly affected the functions of cell growth and death (0.0056 in C group, 0.0054 in CIN group and 0.0053 in CER group, respectively, P = 0.026) and host cell DNA folding, sorting and degradation (0.02375 in C group, 0.0238 in CIN group and 0.02318 in CER group, respectively, P = 0.018), by using the Kyoto Encyclopedia of Genes and Genomes analysis (KEGG, the major public pathway-related database) (**Figure 6**).

### Correlation Analysis and Stratified Statistical Analyses

**Figure 7:**
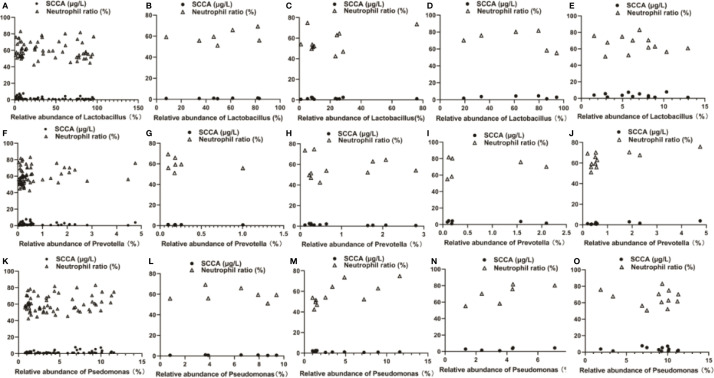
Correlation analysis among Lactobacillus, Pseudomonas, Prevotella and SCCA and Neutrophil ratio using stratified analysis. **(A, F, K)** for the overall level, (B, G, L) for the CINII group, **(C, H, M)** for the CINIII group, **(D, I, N)** for the cervical cancer staged earlier than Ib1 and **(E, J, O)** for the cervical cancer staged later or equal to Ib1. C: Control group, a group of women who have no problem in gynecology. CER: Cervical cancer group, a group of women who were diagnosed a cervical cancer by pathological biopsy. CIN: Cervical intraepithelial neoplasis group, a group of women were diagnosed a by pathological biopsy or cytological test. * means p <0.05; *** means P <0.001; p <0.05 indicates significant difference. ns, no significance.

The correlation between the vaginal microbes and the biochemical test indexes was further studied, and the relative abundance of *Lactobacillus* is negatively correlated with the SCCA (r = −0.2863, p<0.05) and Neutrophil ratio (r = −0.1424, p>0.05). However, *Pseudomonas* has a positively association with SCCA (r = 0.3231, p<0.05) and Neutrophil ratio (r = 0.1833, p>0.05). And *Prevotella* showed the same trend as Pseudomonas [*Prevotella* VS SCCA, r = 0.0589; *Prevotella* VS Neutrophil ratio, r = 0.1743, p>0.05 (shown in **Figures 7A, F, K**)].

The stratified statistical analysis showed that in patients staged earlier than Ib1, *Lactobacillus* was positively correlated with SCCA (r = 0.06858, p > 0.05); and negatively correlated with Neutrophil ratio (r = −0.4340, p > 0.05). *Pseudomonas* had a positively relationship with SCCA (r = 0.5810) and Neutrophil ratio (r = 0.7479, p > 0.05),.And *Prevotella* was negatively correlated with SCCA (r = −0.2835) and positively correlated with Neutrophil ratio (r = 0.1694) (p > 0.05, shown in **Figures 7D, I, N**). However, in patients with a stage later than Ib1, *Lactobacillus* and *Pseudomonas* were negatively correlated with SCCA and Neutrophil ratio (*Lactobacillus* VS SCCA, r = −0.1588, p > 0.05; *Lactobacillus* VS Neutrophil ratio, r = −0.1984, p>0.05. *Pseudomonas* VS SCCA, r = −0.1076, p > 0.05; *Pseudomonas* VS Neutrophil ratio, r = −0.1303, p > 0.05). For *Prevotella*, it is positively correlated with SCCA (r = 0.8404, p < 0.001) and the Neutrophil ratio (r = 0.6470, p < 0.05), (shown in **Figures 7E, J, O**).

For the cervical precancerous lesion patients, CINI group was excluded for only one case. Among the seven patients in the CINII group, the relative abundance of *Lactobacillus* was positively correlated with SCCA (r = 0.4093, p>0.05) and Neutrophil ratio (r = 0.3472, p>0.05), while the relative abundance of *Pseudomonas* and *Prevotella* were respectively negatively correlated with SCCA and Neutrophils (*Pseudomonas* VS SCCA, r = −0.1756, p > 0.05; *Pseudomonas* VS Neutrophil ratio, r = −0.1370, p>0.05. *Prevotella* VS SCCA, r = −0.2538, p > 0.05; *Prevotella* VS Neutrophil ratio, r = −0.2969, p>0.05. Shown in **Figures 7B, G, L**). In the CINIII group, the relative abundances of *Lactobacillus*, *Pseudomonas* and *Prevotella* all showed a negative correlation with SCCA and a positive correlation with Neutrophil ratio (*Lactobacillus* VS SCCA, r = −0.06409, p>0.05; *Lactobacillus* VS Neutrophil ratio, r = 0.4051, p>0.05. *Pseudomonas* VS SCCA, r = −0.5373, p>0.05. *Pseudomonas* VS Neutrophil ratio, r = 0.7053, p<0.05. *Prevotella* VS SCCA, r = −0.6508, p<0.05; *Prevotella* VS Neutrophil ratio, r = 0.03909, p>0.05. Shown in **Figures 7C, H, M**).

## Discussion

As the most prevalent malignant tumor of female reproductive system, although cervical cancer has caused significantly lower mortality rate in recent years due to the improvement of prevention, the incidence is still rising (Reynoso-Noverón et al., 2017). In addition, studies on cervical cancer have long been focused on HPV infection, education and smoking (Koh et al., 2019), but little attention has been paid on vaginal symbiotic microorganisms, which showed a potential connection with cervical cancer in present study.

In this study 90 volunteers were enrolled, and 72 of whom were finally used for the clinical data analysis (**Figure 1**). Our results indicated that a higher HPV infection rate was obtained in CER group compared with C group, which could verify the inter link between HPV and cervical cancer (**Table 1** and **Figure 2**) (Burd, 2003; Castellsagué, 2008). The physiological parameters of blood indicated that cervical cancer had significantly decreased the Lymphocyte ratio and Hemoglobin (P<0.05), while significantly enhanced Neutrophil ratio and the tumor marker SCCA (P = 0.0001) (**Figure 4**). As we all know, Neutrophils and Lymphocytes are the most important immune cells in the blood, and Hemoglobin acts as the “transporter” of oxygen. All of these three play a vital role in human health (Jeronimo et al., 2017; Allen, 2018). Therefore, the increased Neutrophil and reduced Lymphocyte and Hemoglobin indicated a more serious vaginal inflammation as well as a weakened immune system in cervical cancer patients (Zhang H. et al., 2018). In addition, the disorder of the vaginal microbiota leads to infiltration of the cervix in an inflammatory environment, which enhances the recruitment of local immune cells. Studies have shown that the local neutrophil infiltration of cervical tissue in patients with cervical cancer can inhibit the body’s cellular immunity and lead to immune evasion of tumor cells (Movahedi et al., 2008). Therefore, the increased Neutrophil ratio in the peripheral blood may increase the incidence of cervical cancer by accumulating in the cervix. In addition, the high value of SCCA also showed an increased risk for patients to suffer distant metastasis of the tumor (Zhou et al., 2017).

After that, high-throughput technology was used to analysis vaginal microbial composition among groups C, CIN and CER, and a higher α diversity was observed in CER group, and the higher Shannon and Simpson index confirmed a disturbed vaginal microbiota in cervical cancer patients (**Figure 4**) (Mitra et al., 2015). As the increased diversity of vaginal microbes is related to bacterial vaginosis (Xia et al., 2016), preterm birth (Stout et al., 2017), HPV infection (Arokiyaraj et al., 2018) and sexually transmitted diseases (STD) (Eastment and McClelland, 2018), we can hypothesis whether the vaginal microbiota acts as the promoter in the procession of cervical cancer development. When vaginal microbiota were further analyzed at genus level, a lower abundance of *Lactobacillus* and higher abundance of *Prevotella* spp., *Sneathia* spp. and *Pseudomonas* spp. were present in CER group (**Figure 5**). As probiotics, *Lactobacillus* plays an important role in protecting the normal microbial structure in the vagina, and it can also make use of the glycogen in the vaginal mucosa epithelium to get energy and adhere to the vaginal mucosa epithelium to compete against other pathogenic microorganisms (Nasioudis et al., 2017; Niu et al., 2017) by secreting lactic acid, H_2_O_2_, etc. (Medina-Colorado et al., 2017). In addition, studies have shown that *Lactobacillus* can promote vaginal epithelial tissue repair (Takada et al., 2018), thereby reducing HPV infection, and *Lactobacillus* may play a role in the clearance of HPV infection (Castellsagué, 2008). Study, carried out by Soo-Nyung et al. (Kim et al., 2015), have even observed that the HeLa cell line had an accelerated apoptosis response after adding the incubation supernatant of *Lactobacillus casei (L. casei)* to the culture system. This study concluded that *Lactobacillus* may have a killing effect on cervical cancer cells. Pathogen *Prevotella* spp. is a common type of bacteria present in the vagina of adult women, which is closely linked to preterm birth (Stout et al., 2014). Moreover, *Prevotella* can secrete proteases to degrade host antibodies, and can transfer ammonia to the *Gardnerella*, causing excessive secretion of ammonia in the host’s vagina and reducing host mucosal immunity (Pybus and Onderdonk). In addition, *Prevotella* is also confirmed to play an important role in HPV infection and persistence (Di Paola et al., 2017). Therefore, we can conclude that the relative abundance of *Prevotella* is closely related to the occurrence of cervical cancer. For *Pseudomonas* spp., this bacteria strain plays a certain pathogenic role in vaginal inflammation, urinary system infection and respiratory infection. It acts by secreting protease IV (PIV) and inactivating interleukin 22 (IL-22) in order to disrupt the mucosal defense against extracellular pathogens (Bradshaw et al., 2018), providing opportunities for HPV infection, then promote the occurrence of cervical cancer. *Sneathia* spp. is a gram-negative anaerobic bacterium, and previous studies have reported that the increase in abundance of *Sneathia* is related to the occurrence of spontaneous abortion (Seo et al., 2017), non-gonococcal urethritis (Manhart et al., 2013) and bacterial vaginosis (Millar, 2017). In addition, previous studies have confirmed that the relative abundance of *Sneathia* genus is elevated to varying degrees in the vaginal microbiota of patients with cervical precancerous lesions (Łaniewski et al., 2018). Moreover, the KEGG analysis confirmed that the disturbed vaginal microbiota would reduce the cell growth and death, and improve the cell motility, which is consistent with the clinical results (**Figure 6**).

The correlation analysis between vaginal microbes and biochemical test indicators indicated that *Lactobacillus* was weakly negatively correlated with SCCA and Neutrophil ratio at the overall level, while the SCCA value was positively correlated with the distant metastasis of cervical cancer (Zhou et al., 2017), and the higher Neutrophil ratio may also play a role to promote the incidence of cervical malignancy (Movahedi et al., 2008), which indicated that the increased relative abundance of *Lactobacillus* may play a role in inhibiting the distant metastasis and incidence of cervical cancer. On the contrary, the relative abundance of *Pseudomonas* and *Prevotella* is positively related with SCCA and Neutrophil ratio, meaning that *Pseudomonas* and *Prevotella* may play a role in the occurrence and development of cervical cancer (**Figure 7**).

Our results indicated that factors such as the HPV infection, abnormal Neutrophil ratio, Lymphocyte ratio, Hemoglobin and pathological section in cervical cancer patients are very likely to be associated with reduced probiotics *Lactobacillus*, as well as increased Shannon index, Simpson index, pathogens *Prevotella* spp., *Sneathia* spp. and *Pseudomonas* spp. Therefore, vaginal microbiota may play an important role in the occurrence and development of cervical cancer, making it possible that interventions for vaginal microbiota become a potential therapy for invasive cervical cancer. However, the sample size in this study was relatively small, and a larger sample size is required for future systematic studies.

## Data Availability Statement

The datasets generated for this study can be found in the GenBank accession number PRJNA595048.

## Ethics Statement

The studies involving human participants were reviewed and approved by Ethics committee of the second affiliated hospital of Nanchang University. The patients/participants provided their written informed consent to participate in this study.

## Author Contributions

BT and TC designed the study. YPX, YF, WL, FZ, GH, HH, and YFX carried out the experiments. TC and YX analyzed the references and wrote the manuscript. All authors contributed to the article and approved the submitted version.

## Funding

This study was supported by grants from the National Natural Science Foundation of China (grant no.81760729, 82060638), the Key Project of Jiangxi Natural Science Foundation (grant no.20161BBG70218, 20194BCJ22032), and ”double 10-thousand plan“ of Jiangxi Province (innovation and technology professionals as the highend talent).

## Conflict of Interest

The authors declare that the research was conducted in the absence of any commercial or financial relationships that could be construed as a potential conflict of interest.
